# The Interrelationship of Research in the Laboratory and the Field to Assess Hydration Status and Determine Mechanisms Involved in Water Regulation During Physical Activity

**DOI:** 10.1007/s40279-014-0155-0

**Published:** 2014-05-03

**Authors:** Nina S. Stachenfeld

**Affiliations:** Departments of Obstetrics, Gynecology and Reproductive Sciences and Epidemiology and Public Health, The John B. Pierce Laboratory and Yale School of Medicine, 290 Congress Avenue, New Haven, CT 06519 USA

## Abstract

Changes in skin blood and sweating are the primary mechanisms for heat loss in humans. A hot, humid environment concomitant with dehydration limits the ability to increase skin blood flow for the purpose of transferring heat from the body core to skin surface and evaporate sweat to maintain core temperature within safe limits during exercise. Adequate hydration improves thermoregulation by maintaining blood volume to support skin blood flow and sweating. Humans rely on fluid intake to maintain total body water and blood volume, and have developed complex mechanisms to sense changes in the amount and composition of fluid in the body. This paper addresses the interrelationship of research in the laboratory and the field to assess hydration status involved in body water and temperature regulation during exercise. In the controlled setting of a research laboratory, investigators are able to investigate the contributions of volume and tonicity of fluid in the plasma to body water and temperature regulation during exercise and recovery. For example, laboratory studies have shown that tonicity in a rehydration beverage maintains the thirst mechanism (and stimulates drinking), and contributes to the ongoing stimulation of renal fluid retention hormones, ultimately leading to a more complete rehydration. Research in the field cannot control the environment precisely, but these studies provide a natural, ‘real-life’ setting to study fluid and temperature regulation during exercise. The conditions encountered in the field are closest to the environment during competition, and data collected in the field can have an immediate impact on performance and safety during exercise. There is an important synergy between these two methods of collecting data that support performance and protect athletes from harm during training and improve performance during competition.

## Introduction

When assessing an individual’s hydration status, there is no one total body water (TBW) that represents euhydration; determinations need to be made of body water fluctuations beyond a range that have functional consequences [1]. Ideally, the hydration biomarker should be sensitive and accurate enough to detect body water fluctuations of approximately 3 % of TBW (or water content change sufficient to detect fluctuations of ~2 % body weight for the average person). In addition, the biomarker should also be practical (time, cost, and technical expertise) to be used by individuals and coaches [2].

## Importance of the Problem

Mortality as a result of overheating during heat waves in the summer months exceeds that due to lightning, rain, floods, hurricanes, and tornadoes combined [3]. During physical activity metabolic heat production from the working muscles increases core temperature. When exercising in the heat, the risk of overheating is exaggerated because environmental factors, such as water vapor pressure and airflow, can limit the ability to move heat from the body core to the surface. In addition, non-thermal factors, such as plasma osmolality [4] and central hypovolemia [5], modulate skin blood flow so changes in these factors can also limit sweat evaporation and the maintenance of core temperature within safe limits. Hyperthermia occurs when heat dissipation from sweating cannot keep up with the heat generated from the working muscles [6]. Depending on intensity and environmental factors, exercise-related heat production can be as much as 15–20 times greater than at rest, and would raise core body temperature by 1 °C every 5 min if no heat were dissipated [7].

Exercise duration and intensity, environmental conditions, fitness (maximal oxygen consumption), hydration status, and heat acclimation all affect thermoregulation during exercise [8]. Certain medications and overall health can also play a role in thermoregulation during exercise if they impact cardiovascular function or sweating. Active people, who are not professional athletes, face unique risks for heat illnesses because they exercise unsupervised (often alone), they may become dehydrated if they do not practice a hydration plan, their fitness level is generally lower than that of athletes, they may be less acclimatized to heat, and the risk of hyperthermia is exaggerated in individuals with a body mass index >27 kg/m^2^ [8–18]. For example, studies in miners, who are exposed to significant heat challenges on a daily basis, have found that the primary variables that affect exhaustion during work are fitness, body mass index >27 kg/m^2^, dehydration, air temperature >33 °C, and air velocity <2.0 m/s [19–22].

In humans, skin blood flow increase during heat exposure enhances conductive and convective heat loss from the body and also augments heat transfer from the body core to the skin surface. This process occurs even in a hot environment in which the atmospheric temperature is higher than the skin surface. Cutaneous vasodilation thus promotes heat transfer from the body core to the skin, contributing to heat dissipation in hyperthermia. Moreover, in order to support sweating and heat dissipation, fluid moves from the blood stream to the skin where evaporation cools the body. These systems are sensitive to baroreceptors [23], so maintaining blood volume is an essential component of thermoregulation during exercise. In turn, continuing loss of fluid volume from the body can lead to dehydration, and poses a significant risk of hyperthermia if not replenished. Humans rely on fluid intake to maintain TBW and blood volume, and have developed complex mechanisms to sense changes in the overall amount of fluid in the body and its composition. The laboratory is ideal for investigating these mechanisms and how they are impacted by the personal and environmental conditions described earlier. In addition, markers that can be used in the field are developed through laboratory experimentation. Field studies that apply laboratory data may improve athletic performance and safety for both amateur and professional athletes, and data from both types of studies have indicated important synergy between the two [24, 25]. This review first describes some of the mechanisms involved in fluid regulation and how they function during exercise. It then describes how dehydration and exercise-associated hyponatremia (EAH) occur when the body is pushed to extremes and is unable to employ the mechanisms designed to maintain hydration and tonicity appropriate to support human function. Finally, the review describes the respective contributions of laboratory and field studies to athletes’ training safety and improving performance.

## Overview of Thirst and Water Regulation in Humans

**Fig. 1:**
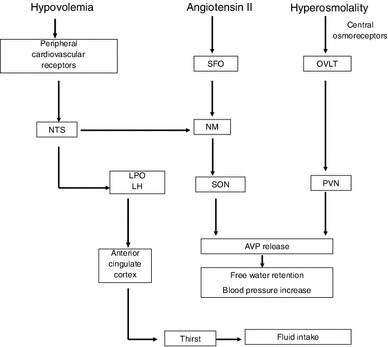
Schematic diagram of central regulation of body fluid regulation in response to acute changes in sodium and volume. Reproduced from Stachenfeld [27], with permission. *AVP* arginine vasopressin, *LH* lateral hypothalamus, *LPO* lateral preoptic nucleus, *NM* median preoptic nucleus, *NTS* nucleus of the solitary tract, *OVLT* organum vasculosum of the lamina terminalis, *PVN* paraventricular nucleus, *SFO* subfornical organ, *SON* supraoptic nucleus

Thirst sensation and water status are regulated by the central nervous system, which receives signals relevant to hydration status from both central and peripheral pathways (Fig. 1) [26, 27]. The organum vasculosum of the lamina terminalis (OVLT), a circumventricular organ located outside the blood–brain barrier in the anteroventral part of the third ventricle in the brain, is a principal component of the fluid regulation pathway sensitive to changes in plasma osmolality. Changes in peripheral plasma osmolality associated with changes in body water or sodium status are sensed by neurons in the OVLT, transmitted to the hypothalamus and stimulate thirst sensation, drinking and arginine vasopressin (AVP) release. Another important pathway initiating drinking operates by means of the median preoptic nucleus (NM), which responds to volumetric changes sensed by atrial baroreceptors. Therefore, with dehydration, changes in osmotic content and volume are sensed by peripheral receptors to stimulate either hormone actions (release or suppression) or drinking behavior (drinking or to stop drinking). The subfornical organ (SFO) and the nucleus of the solitary tract, structures at the center of sodium appetite and thirst regulation, also utilize the NM mechanism. In the latter mechanism, changes in total body volume are initiated by the kidneys, and stimulated by angiotensin outside the blood–brain barrier. Angiotensinergic thirst stimulation through the SFO also plays a role in peripheral thirst signaling by sending neural messages to the NM, which then initiates volumetrically controlled thirst and drinking (Fig. 1) [26, 27].

During dehydration or body water loss, angiotensin also stimulates the secretion of fluid-regulating hormones by the pituitary and adrenal glands, and increases or maintains blood pressure. Neurons in the paraventricular and supraoptic nuclei, both located in the hypothalamus, control AVP release by the posterior pituitary, so both the paraventricular and supraoptic nuclei represent important structures involved in the control of water and sodium regulation. AVP controls renal free water regulation, but is also one of the most powerful vasoconstrictors in the body, and is also a key hormone in blood pressure regulation. Finally, the anterior cingulate cortex is involved in the relay of neural signals between the right and left hemispheres of the brain and is involved in decision making, and plays an important role in sensing thirst and initiating drinking [26]. For long-term control of fluid and sodium regulation and blood pressure, the kidney is sensitive to body water status and will excrete or retain sodium and water as needed.

## Measurement of Cognitive Thirst Sensation in Humans

Visual analog rating scales have been used to assess thirst perceptions in humans [28] during exercise-induced dehydration [29], exercise-associated hyponatremia [30], and hypertonic saline infusion (HSI) [31, 32]. The subjects respond to the question “How thirsty do you feel right now?” by marking on a line approximately 180 mm long with intersecting lines anchored at 0 mm for “not at all” and at 125 mm for “extremely thirsty.” This scale has been used extensively for psychophysical assessments in both older and younger individuals and corresponds to physiological determinants of thirst, such as plasma osmolality [32, 33]. These scales are also used to measure dry mouth, stomach fullness, and flavor perception to provide more nuanced information with regard to drinking behavior [28]. The earlier scales used paper and pencils, and more recent scales are computerized and therefore more accessible and convenient for study subjects and investigators in both laboratory and field environments.

### Laboratory Research

**Fig. 2:**
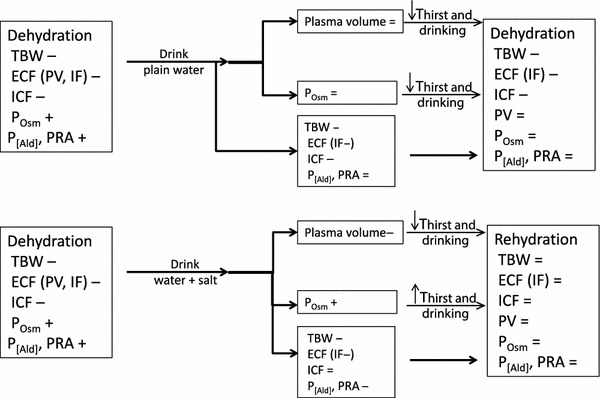
Schematic diagram comparing recovery from dehydration with plain water (*top*) to recovery from dehydration with water and salt (*bottom*). *ECF* extracellular volume, *ICF* intracellular volume, *IF* interstitial volume, *P*_[*Ald*]_ plasma aldosterone concentration, *P*_*Osm*_ plasma osmolality, *PRA* plasma renin activity, *PV* plasma volume, *TBW* total body water

Research in the laboratory has the advantage of carefully controlled conditions to study mechanisms involved in fluid and electrolyte regulation. For example, a seminal series of studies demonstrated the advantage of adding sodium to a rehydration protocol [34–36]. When humans drank plain water during exercise or while recovering from exercise the water was preferentially retained in the plasma even though the fluid in the interstitial and cellular spaces had not been restored [36]. The effect of this preferential restoration of water into the plasma is the suppression of the receptor activity involved in thirst and fluid regulation in the brain and kidney, as described earlier (Fig. 1). In a sense, the early plasma volume restoration ‘fools’ the systems involved in stimulating thirst and controlling fluid retention into suppressing thirst and fluid-regulating hormones, followed by reduced drinking and renal water retention despite incomplete TBW restoration. This physiological phenomenon has been termed “involuntary dehydration” [37] or “voluntary dehydration” [38]. This series of laboratory studies [34–36] also demonstrated that sodium ingestion during recovery from dehydrating exercise provided osmotic stimuli that continued to stimulate thirst and stimulate fluid retention hormones [34–36], resulting in a more complete restoration of TBW (Fig. 2). Adding sodium thus increased osmotic pressure in the plasma and maintained the thirst stimulus. The greater fluid ingestion enabled other body fluid compartments, as well as TBW, to achieve a more complete restoration. These investigators also demonstrated the important role that the sodium-regulating hormones aldosterone and renin play in the fluid restoration process following dehydrating exercise [35]. The measurements performed in those studies required a controlled laboratory environment for frequent blood collection and sophisticated equipment, but laid the foundation for much laboratory and fieldwork to follow.

Later studies determined mechanisms related to osmotic thirst and hormone regulation in humans, and examined these mechanisms in different populations such as women and in aging adults. In all groups, plasma osmolality is a strong linear predictor of both thirst and plasma AVP concentration (P_[AVP]_) (*r* ≥ 0.90) [31–33, 39]. A steeper slope within these relationships demonstrates heightened sensitivity of central osmoreceptors that stimulate the cognitive sensation of thirst and cause the release of AVP from the hypothalamus and posterior pituitary. In contrast, a flatter slope can indicate diminished osmotic function, as is often seen in aging populations. In the laboratory, it is possible to examine osmotic regulation of thirst and/or AVP under a number of different conditions that stimulate either osmotic or volume receptors [31, 32, 39]. Dehydration, induced by sweating during exercise or heat stress, leads to simultaneous increases in plasma osmolality and serum sodium concentrations because sweat is hypotonic to plasma, making water move quickly out of the vascular space. The concomitant increase in plasma osmolality with plasma volume loss (i.e., a hyperosmotic, hypovolemia) stimulates thirst and AVP through both tonic and volume receptor mechanisms (Fig. 1). The use of graded dehydration to study acute thirst or hormonal responses to increasing sodium concentration in the blood is important because it is a primary physiological response to exercise. Moreover, comparing differences in slope or threshold in this relationship can also indicate basic differences in function as they do when comparing two phases of the menstrual cycle or sex differences, for example. However, a weakness of the dehydration protocol is that exercise-induced dehydration does not isolate the independent contributions of volume and osmoreceptors to the overall thirst or AVP responses because both are changing simultaneously [29, 34, 40]. This weakness can assume special importance if a goal of the experiment is to determine the cause of an impairment found in a special population.

In order to examine specific osmotic mechanisms involved in the regulation of thirst and body water, HSI (3 % NaCl) is used to examine osmoreceptor input into thirst sensation and AVP responses [33]. HSI is a powerful method to examine mechanisms controlling thirst and AVP regulation in humans because the rapid increase in plasma sodium (and plasma osmolality) induces rapid and linear thirst and AVP responses. HSI causes plasma osmolality increases of 16–20 mOsmol/kg water during a 2-h infusion [33], so is an excellent method to compare plasma osmolality–P_[AVP]_ and plasma osmolality–thirst slopes and intercepts across populations and conditions. During and following the infusion period, urine samples are collected so the primary renal responses to AVP (free water and osmotic clearances) are determined. This type of laboratory study enables a precise examination of the osmotic regulation of thirst and AVP as well as renal fluid regulation. A limitation for this type of study is that HSI induces a large intravascular fluid expansion (~20 %) concomitant with the rise in plasma osmolality as water is drawn from cells in response to the increased osmotic pressure in fluid surrounding them (i.e., a hyperosmotic, hypervolemia). HSI thus induces opposing inputs from osmotic and volume reflexes, although clearly the osmotic stimulus prevails as indicated by the large increases in thirst ratings and P_[AVP]_ [32]. While HSI induces greater increases in plasma osmolality and plasma volume than that encountered during a typical exercise bout, these experiments have proved important to our understanding of mechanisms regulating P_[AVP]_ and thirst and are consistent with findings during dehydration [29]. For example, it was possible to study sex hormone effects on these regulatory mechanisms more closely with HSI [29, 32, 41]. Moreover, the use of this technique is helpful in understanding osmotic regulation in older populations who may not be able to exercise in the heat for long periods of time [32].

Thermoneutral head-out water immersion combined with dehydration has been used to dissect the independent influences of the osmotic and volume stimuli during dehydration [31, 42]. In such studies subjects are dehydrated with exercise in the heat (~2 % body water loss) followed by overnight water deprivation. The following morning, the subjects (still ~2 % dehydrated) sit in a thermoneutral water tank with only their head out of the water. During this thermoneutral head-out water immersion, approximately 700 mL of water is driven into the intrathoracic space from hydrostatic pressure on the blood vessels, leading to increases in cardiac filling pressure [43]. The subjects are thus still dehydrated from their exercise bout/water deprivation because TBW remains low and plasma osmolality remains high, but central blood volume, stroke volume, and fluid-regulating hormones are restored to pre-exercise levels [31, 42]. This central volume expansion activates cardiac volume (stretch) receptors, which attenuates thirst and drinking despite continuing dehydration and elevations in plasma osmolality [31, 42]. These studies are used to define the relative contribution of volume compared with osmo-receptors to thirst and AVP responses [42], and to determine changes in these functions with aging [31].

A final example of a laboratory study important for future field investigations is the study of EAH. Finishing a marathon or other endurance event typically results in approximately 2–3 % loss in TBW concomitant with increases in plasma sodium concentration of approximately 5–7 mEq/L. In EAH, some athletes (1–13 %) reduce sodium concentration by 5 mEq/L or greater relative to pre-race values at the end of endurance exercise [44, 45], usually the result of excess hypotonic fluid ingestion accompanied by significant water retention. Mechanisms contributing to EAH have proved difficult to examine because the studies in which athletes have EAH following racing are retrospective; that is, athletes are examined and grouped after they have become hyponatremic during a race but are not grouped before the race with the purpose of measuring drinking behavior or other variables associated with EAH in the field. In order to determine prospectively specific risk factors and variables that increase the risk of EAH, subjects with a history of hyponatremia were recruited into the laboratory. Both controls (subjects with no hyponatremia history) and ‘high risk’ EAH subjects performed long-term exercise with fluid (water) intake precisely controlled [30]. Both groups of subjects drank 8 mL/kg of their body mass in water in addition to replacing any water losses (measured sweat, urine) during exercise. Those studies demonstrated that water retention, not sodium loss, was the primary contributor to the lower exercise serum sodium concentration (S_[Na+]_) in women at risk of EAH. However, sex hormone interventions suggested that sodium loss might be a more important factor in EAH in women during increased progesterone exposure.

Whereas HSI, immersion, and EAH studies do not mimic any natural physiological state, these protocols were needed to understand mechanisms underlying the control of thirst and overall body fluid regulation in humans. The goal of such studies is to advance scientific knowledge and provide the basis for research that can take place in the field to guide hydration decisions for active individuals and reduce failures of thermoregulation that lead to clinical heat illnesses (heat exhaustion, heat stroke), and to improve performance.

### Field Research

In the laboratory, with few exceptions such as running, rowing or cycling [46], conditions can only be modeled to be as close as possible to the actual sport [47]. In field research, the scientist has the advantage of studying responses in conditions that the athlete will experience during competition or during training. These conditions can be challenging (but not impossible) to reproduce in the laboratory. In addition, in field studies the athletes are performing their own specific activity with the equipment they use in competition. The information derived from a carefully conducted field study should thus easily be applied to the individual athlete, and is therefore immediately valuable. However, standard and precise laboratory measurements of hydration, such as plasma osmolality measurements, are not available to most athletes or active people, and are cumbersome to use in the field even by professionals [48]. Precise measurements of blood and plasma volume and fluid regulation hormones require blood sampling, so these methods are also impractical for individuals and professionals in a field environment. Controlling intensity is not practical during studies conducted during competition, but recent technology is improving the ability to measure power output and oxygen consumption in the field. Portable devices, which can estimate oxygen consumption and caloric expenditure using a triaxial accelerometer and a barometer, have been developed for walking uphill [49], while other more simplified devices that estimate overall movement have appeared on the market. It is projected that these devices can be applied to any physical activity, making field estimates of intensity more precise.

Carefully controlled conditions and outdoor courses can also provide important information on hydration (when experiments are conducted under similar environmental conditions) while intensity is controlled using physiological measures such as heart rate [50, 51]. Individual athletes and investigators can determine hydration with the use of biomarkers that are non-invasive and simple, and have validity if care is taken to control as much of the environment as possible [50]. For example, hydration status can be determined by participants examining the first morning void for color to indicate concentration. While using color can be subjective, scales have been developed for this purpose [52], and this marker can be valuable to determine changes in hydration status [50, 53]. Combining urine examination with measurement of first morning body weight can provide an indication of any significant changes in hydration status, and using changes in body weight together with urine color as biomarkers provides significantly more insight than using them individually [2]. During training, the athlete participant should establish a baseline body weight by using first morning weight over a series of days. For long-term studies, investigators can then compare post-exercise or daily body weights of the athletes to improve the accuracy of body weight as a marker of hydration status. An important caveat is that these urine and body weight biomarkers are only valid during periods of stable fluid status. During drinking or rehydration periods urine may appear light while the individual remains dehydrated (due to involuntary dehydration, as described above). In this circumstance, although urine volume increases and appears dilute, body weight will not be entirely restored.

More simple solutions include counting urine duration in seconds to estimate volume if collection is not viable, the athletes can estimate their own fluid intake when bottles or cups are supplied to them, and body weights can be measured before, during and after the event or training sessions to determine sweating volume (consider 1 mL of sweat loss represents a 1 g loss in body mass because the specific gravity of sweat is 1.0 g/mL). While there are respiratory water losses during exercise, these losses are small, so cause only a minor (insignificant) overestimate of sweating loss. During races the investigator can determine and record environmental conditions on the race day and track them over time. In order to measure physiological variables, minimally intrusive heart rate, temperature, and blood gas monitors are also available. Other measurements described within laboratory studies that measure thirst and other sensations related to hydration status can easily be employed in the field by coaches, or even by the individual athlete. Scales that measure ratings of comfort and perceived exertion are also valuable tools to monitor hydration status, intensity and temperature responses to exercise. In addition to these basic techniques, a number of elegant systems have been developed for use in the field to determine hydration [48]. These systems include portable refractometers to determine urine specific gravity, as well as portable urine conductivity analyzers [54] and arm radio frequency analyzers [55]. These portable instruments accurately predict urine osmolality and body mass loss, respectively [48].

**Fig. 3:**
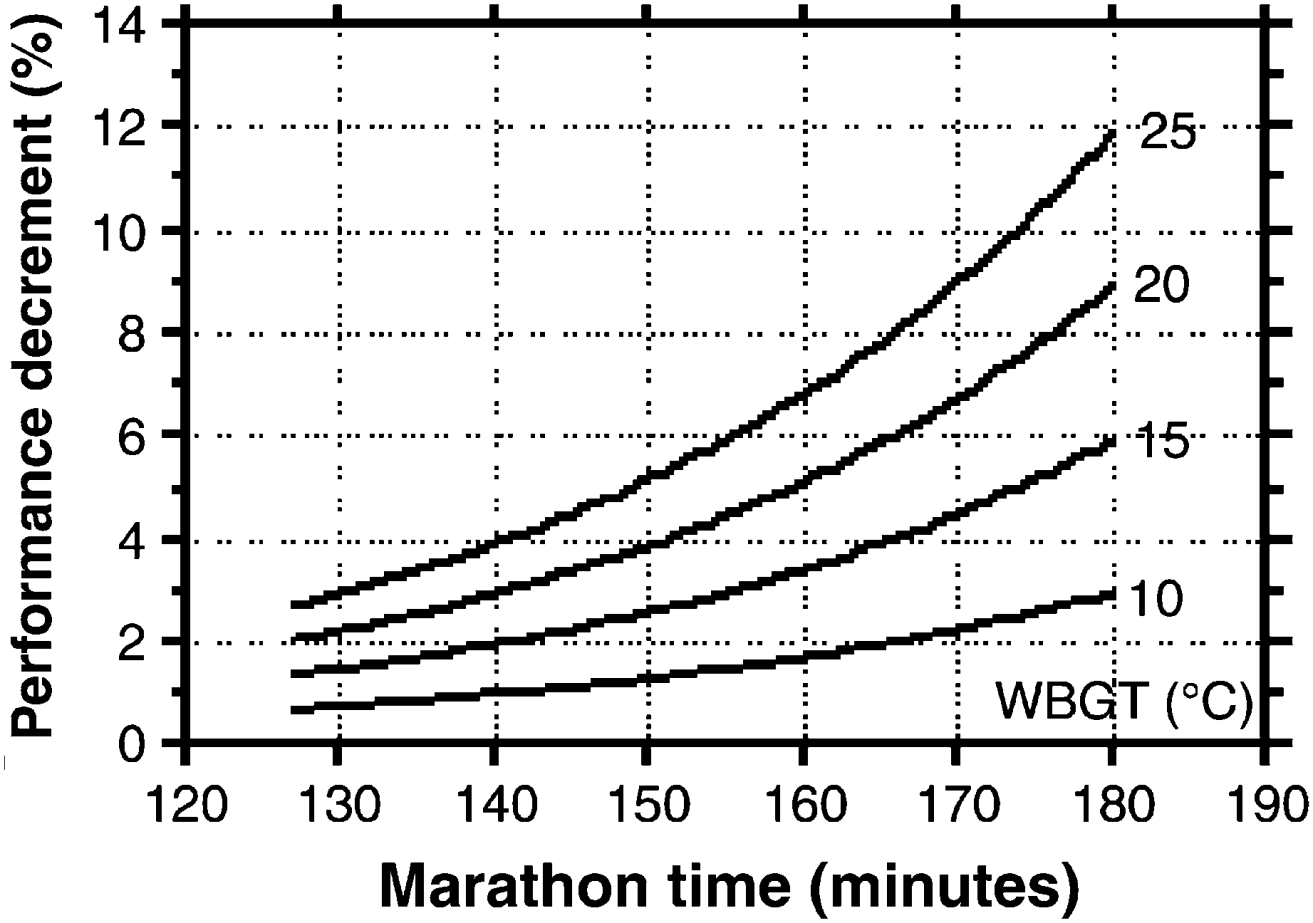
Nomogram indicating the predicted performance decrement (%) based on projected marathon finishing time (*x*-axis) with increasing wet-bulb globe temperature (WBGT). WBGT is a composite temperature used to estimate the effect of temperature, humidity, and solar radiation on humans. Data are based on results from seven separate marathons and include data based on elite and recreational runners. Reproduced from Ely et al. [56], with permission

Another method of field research is using data assembled by observation. Ely et al. [56] provide an interesting example. These investigators examined the impact of different temperature and air quality conditions on marathon performance. In order to obtain enough data, the investigators gathered marathon results and weather data from seven different marathons among cities with widely varying weather conditions. They examined data spanning 10–36 years from these marathons and compared the top three male and female finishers as well as the 25th-, 50th-, 100th-, and 300th-place finishers with course records. These data were then analyzed according to the different weather conditions across the marathons and years. While they were unable to control marathon conditions or performance characteristics, they collected enough data (*n* = ~136) so they could divide temperature data into quartiles and compare performance across them. That study demonstrated that hot and humid weather had a strong detrimental effect on marathon performance, which was exaggerated in women and in those with longer running times (Fig. 3). As the wet bulb globe temperature increased from 10 to 25 °C, elite competitors lost approximately 2 % off their best marathon times (2–3 min, enough to cost them victory). However, finishers with times >3 h could finish as much as 10 % slower.

## Conclusions

Laboratory and field research both have strengths and limitations. Laboratory research is best suited for examining questions that require stringent environmental and physiological control, and are useful for defining mechanisms for physiological responses. Laboratory research is very much dependent on field research to supply research questions and challenges. Field research is closest to the athlete, and data collected in the field are most likely to have an immediate impact on improvements in performance and safety during exercise. However, field studies can monitor, but not control, environmental conditions, such as the temperature and humidity, which can be variable across research days, practice or training days, and competition. Moreover, performing interventional studies in the field is challenging because interventions for the purpose of research may impact performance. Importantly, data from field studies show a remarkable consistency with body fluid and temperature data collected in the laboratory [24, 25]. Therefore, despite differences in methodology between the laboratory and field, these two disciplines work synergistically to investigate physiological responses during physical activity.
